# Clinical correlates of new-onset and persistent suicidal ideation in adolescents with major depressive disorder

**DOI:** 10.3389/fpsyt.2025.1655037

**Published:** 2025-09-10

**Authors:** Yun Zhang, Lewei Liu, Haojie Fan, Xi Zhang, Lei Xia, Huanzhong Liu

**Affiliations:** ^1^ Department of Psychiatry, The Fourth Affiliated Hospital of Anhui Medical University, Hefei, Anhui, China; ^2^ Department of Psychiatry, School of Mental Health and Psychological Sciences, Anhui Medical University, Hefei, Anhui, China

**Keywords:** suicidal ideation, major depressive disorder, adolescents, predictive factors, longitudinal study

## Abstract

**Background:**

Suicidal ideation (SI) is common in adolescents with major depressive disorder (MDD). SI not only poses a serious threat to the patient’s life safety, but also significantly hinders the process of psychological recovery and the restoration of social functioning. However, there is still a relative lack of longitudinal studies on the factors influencing SI in adolescents with MDD. Therefore, this study aimed to explore the longitudinal trajectory of SI in adolescents with MDD and to identify the relevant influencing factors.

**Methods:**

This study included 122 adolescents with MDD. At baseline and one-year follow-up, patients were assessed for SI. Based on the assessment results, patients were divided into SI group and non-SI group. In addition, the standardized questions and the Center for Epidemiological Studies Depression scale (CES-D), the Childhood Trauma Questionnaire (CTQ), and the Toronto Alexithymia Scale (TAS-20) were used to evaluate non-suicidal self-injury (NSSI), depressive symptoms, childhood maltreatment (CM), and alexithymia. Logistic stepwise regression analyses were employed to identify factors independently associated with SI in adolescents with MDD.

**Results:**

In adolescents with MDD, the prevalence of SI was 68.0%. At the follow-up period, the prevalence of persistent suicidal ideation (PSI) was 19.7%, and the prevalence of new-onset SI was 20.5%. Regression analyses showed that single-child family (*OR* = 3.969, 95%*CI:* 1.227 - 12.839, *P* = 0.021), TAS-20 score (*OR* = 1.091, 95%*CI*: 1.006 - 1.184, *P* = 0.035), and difficulties identifying feelings (*OR* = 1.134, 95%*CI:* 1.000 - 1.287, *P* = 0.050) were risk factors for PSI. NSSI (*OR* = 4.552, 95%*CI*: 1.488 - 13.921, *P* = 0.008) and positive affect (*OR* = 1.424, 95%*CI*: 1.125-1.804, *P* = 0.003) were risk factors for new-onset SI.

**Conclusion:**

Adolescents with MDD have a high risk of PSI, and new-onset SI should not be ignored. Factors such as single-child family, alexithymia, NSSI, and reduction of positive affect significantly affect the occurrence and persistence of SI. Therefore, early intervention targeting these factors is important to reduce the risk of adolescent suicide and improve mental health outcomes.

## 1 Introduction

Major depressive disorder (MDD) is a mental illness characterized by significant and persistent low mood, which is particularly common in adolescents (1). A study indicated that the point-in-time prevalence and lifetime prevalence of MDD in global adolescents were 8.0% and 19.0%, respectively (2). MDD not only had a serious impact on the physical and mental health of adolescents, but also significantly increased their risk of suicide (3). Suicidal ideation (SI) is a common psychological phenomenon in adolescents with MDD. It refers to the psychological state in which an individual has the idea of ending their life but has not yet taken any actual action. A cross-sectional study showed that the prevalence of SI in adolescents with MDD was as high as 75.7% (4). This indicates that the prevalence and severity of SI in this group cannot be ignored. However, although cross-sectional studies reveal a high incidence trend of SI, it is difficult to elucidate its longitudinal progression. Therefore, in recent years, scholars both domestically and internationally increasingly focused on the persistent characteristics of SI. They referred to the phenomenon where individuals reported SI at baseline assessment and again at follow-up as persistent suicidal ideation (PSI) (5, 6). A study showed that PSI was a more effective predictor of suicide attempt than single-point-in-time reports of SI (7). A recent data further showed that the probability of a suicide attempt for individuals with PSI was 13.8%, compared to 1.8% among individuals who reported only a single episode of SI (8). In addition, adolescents with PSI were at significantly higher risk of attempting suicide in adulthood (9). Therefore, SI, particularly PSI, serves as an important predictor of future suicide risk in adolescents.

Currently, interventions for SI in patients with MDD were still largely focused on the acute management phase after the onset of symptoms. Although treatments such as modified electroconvulsive therapy (10), subnarcotic doses of esketamine (11, 12), and circadian rhythm modulation (13)could indirectly ameliorate SI through the rapid alleviation of depressive symptoms, particularly in patients with treatment-resistant depression (14), their efficacy for use in preventing the onset of SI in the adolescent population had not been systematically evaluated. Therefore, investigating the potential influencing factors of SI in adolescents with MDD was crucial for early identification, prevention and optimized treatment in this population.

The emergence of SI is a complex psychological process, which involves the interaction of multiple factors, including psychological factors, socio-environmental factors, and deficits in individual coping capacity. Among these factors, the severity of depressive symptoms was found to be the most important predictor of SI, and the two are significantly correlated (15). A longitudinal study further revealed that improvements in depressive symptoms often preceded reduction in SI, suggesting that depressive symptoms may be an important driver of SI (16). It was worth noting that a low level of positive affect might be an independent and important mechanism, especially prominent in SI and behavior among adolescents (17). For example, the absence of positive affect was found to be an independent risk factor for SI, which may be related to an individual’s psychological resilience and emotional regulation ability (18). Further research indicated that the absence of positive affect could more specifically predict the risk of suicide than negative emotions, which was particularly important for the assessment of suicide risk among adolescents (19).

On the other hand, research has shown that childhood maltreatment (CM) was significant risk factors for SI, with emotional abuse having an independent predictive effect on SI (20). CM not only directly increases the risk of SI but also indirectly promotes its development by exacerbating emotional regulation difficulties and depressive symptoms (21). Similarly, alexithymia, as a psychological trait, had a significant positive correlation with SI (22, 23). Specifically, a 12-month prospective follow-up study found that, after controlling for baseline depression levels, changes in alexithymia characteristics were independently associated with the occurrence or reduction of SI (24). Furthermore, an exploratory study based on daily clinical practice indicated that alexithymia is one of the important predictors of SI during the first episode of depressive disorder (25). Further analyses indicates that specific sub-dimensions of alexithymia, such as difficulty in identifying feelings, are more significantly associated with SI. For instance, a cross-sectional study found that patients with SI scored significantly higher on the subscale of difficulty in identifying feelings, suggesting that difficulty in recognizing one’s own emotions may directly increase the risk of suicide (26).

In addition, non-suicidal self-injury (NSSI) is considered one of the important risk factors for SI. It reflects the impulsivity and self-harm tendencies of individuals in response to negative affect (27). A prospective longitudinal study among German adolescents showed that individuals with NSSI behaviors at baseline had a significantly higher risk of developing SI or engaging in suicide attempt two years later, with a two-fold increase (28). Therefore, early and accurate identification and effective intervention of these underlying factors are crucial for preventing suicide. It also provides a key entry point for exploring the persistence of SI.

However, most current studies on SI in adolescents with MDD use cross-sectional designs, which make it difficult to reveal the dynamic process of SI. The aim of this study is to systematically evaluate the dynamic changes of SI in adolescents with MDD through a longitudinal study method, and to explore in depth the influencing factors related to persistent and new-onset SI, thereby providing new longitudinal evidence for the mechanism interpretation of SI and the development of intervention programs.

## 2 Methods

### 2.1 Study design and participants

This study employed a prospective research design. From January 2021 to December 2023, we conducted a questionnaire survey in adolescents with MDD who visited the Fourth Affiliated Hospital of Anhui Medical University. The survey was administered at both the baseline assessment and the one-year follow-up. Inclusion criteria were as follows: (1) age between 12 and 18 years; (2) fulfillment of the diagnostic criteria for MDD according to the Diagnostic and Statistical Manual of Mental Disorders, fifth edition (DSM-5); (3) no prior treatment or receipt of only conventional antidepressant medication (e.g., SSRIs, SNRIs) before enrollment, with no other interventions, such as electroconvulsive therapy; and (4) ability to understand and complete the entire study process. Exclusion criteria included: (1) current or previous diagnosis of other mental disorders (e.g., schizophrenia, bipolar disorder); (2) comorbid serious infections, autoimmune diseases, or other severe physical illnesses; (3) change in diagnosis after reassessment according to DSM-5 criteria during the follow-up period; and (4) inability to complete the assessment or cooperate with follow-up visits.

To ensure the accuracy of the measurement results, all data collection and scale assessment work were completed by two clinicians who had undergone strict consistency training. Before the subjects underwent each assessment, the assessors gave them detailed explanations to ensure that the subjects could fully understand the meaning of the questions. This study was reviewed and approved by the Ethics Committee of the Fourth Affiliated Hospital of Anhui Medical University (Approval Number: 202009-KYXM-04). Prior to the commencement of the study, detailed information regarding the study procedures was provided to all participants and their legal guardians, who were required to sign written informed consent forms.

### 2.2 Measuring instruments

#### 2.2.1 Socio-demographic characteristics

A self-designed questionnaire was used to collect socio-demographic information from all enrolled patients, including gender, age, body mass index (BMI), age at onset, duration of illness, single-child family, NSSI and antidepressant use. NSSI was evaluated according to the criteria in DSM-5 (29).

#### 2.2.2 SI

The Beck Suicidal Ideation Scale (BSI) was used to assess the SI of patients in the recent week (30). BSI comprises 19 items and employs a 3-point scoring system. The study used the first five items of this scale to screen patients for SI. If both items 4 and 5 were scored as 0, then SI was considered to be absent; otherwise, SI was considered to be present (31, 32).

#### 2.2.3 Depressive symptoms

In this study, the Center for Epidemiological Studies Depression Scale (CES-D) was employed to assess the depressive symptoms of patients over the past week (33). CES-D is a self-assessment questionnaire comprising 20 items. Each item is rated on a 4-point scale, ranging from “0 = None” to “3 = Almost every day”. The scale is divided into four factors: depressed affect, positive affect, somatic complaints, and interpersonal difficulties (34). The total score ranges from 0 to 60 points, with higher scores indicating more severe depressive symptoms. CES-D demonstrated good reliability and validity among Chinese adolescents (35).

#### 2.2.4 Childhood trauma

In this study, the Childhood Trauma Questionnaire (CTQ) was employed to assess the trauma experienced by the research subjects during their childhood (36). The questionnaire comprises 28 items, each rated on a 5-point scale ranging from “1 = never” to “5 = always”. It covers five dimensions: emotional abuse, physical abuse, sexual abuse, emotional neglect, and physical neglect. The total score of CTQ ranges from 25 to 125 points, with higher scores indicating more severe trauma. The Chinese version of the CTQ has shown good reliability and validity with adolescents in previous studies (37).

#### 2.2.5 Alexithymia

In this study, the Toronto Alexithymia Scale (TAS-20) was employed to assess individuals’ difficulties in recognizing and expressing emotions (38). This questionnaire contains 20 items. Each item is scored on a 5-point scale, ranging from “1 = completely disagree” to “5 = completely agree”, and is divided into three subscales: difficulty identifying feelings, difficulty describing feelings, and externally oriented thinking. The total score range of TAS-20 is 20 to 100 points. The higher the score, the more severe the alexithymia.

### 2.3 Statistical analysis

All statistical descriptions and analyses were conducted using SPSS 23.0. Continuous variables were presented as mean ± standard deviation (SD) or median (quartiles) [M (P_25_, P_75_)]. For data that conformed to the normal distribution, the independent samples t-test was used for intergroup comparisons. If the data did not conform to the normal distribution, the Mann-Whitney U test was employed. Categorical variables were expressed as frequency (%), and the chi-square test was used for comparisons between the two groups. The independent variables included socio-demographic and clinical variables that varied between groups, while the dependent variable was SI. Logistic stepwise regression analyses was performed using the “Forward: LR” method to identify the factors independently associated with SI in adolescents with MDD, and to calculate the corresponding odds ratios (OR) and 95% confidence intervals (CI). Model 1 included socio-demographic variables with intergroup differences and total scale scores with intergroup differences. Model 2 included socio-demographic variables with intergroup differences and subscale scores with intergroup differences. All statistical tests were two-tailed, and a *P*-value of ≤ 0.05 was considered statistically significant.

## 3 Results

### 3.1 Comparison of clinical characteristics between the SI group and the non-SI group in adolescents with MDD

**Figure 1:**
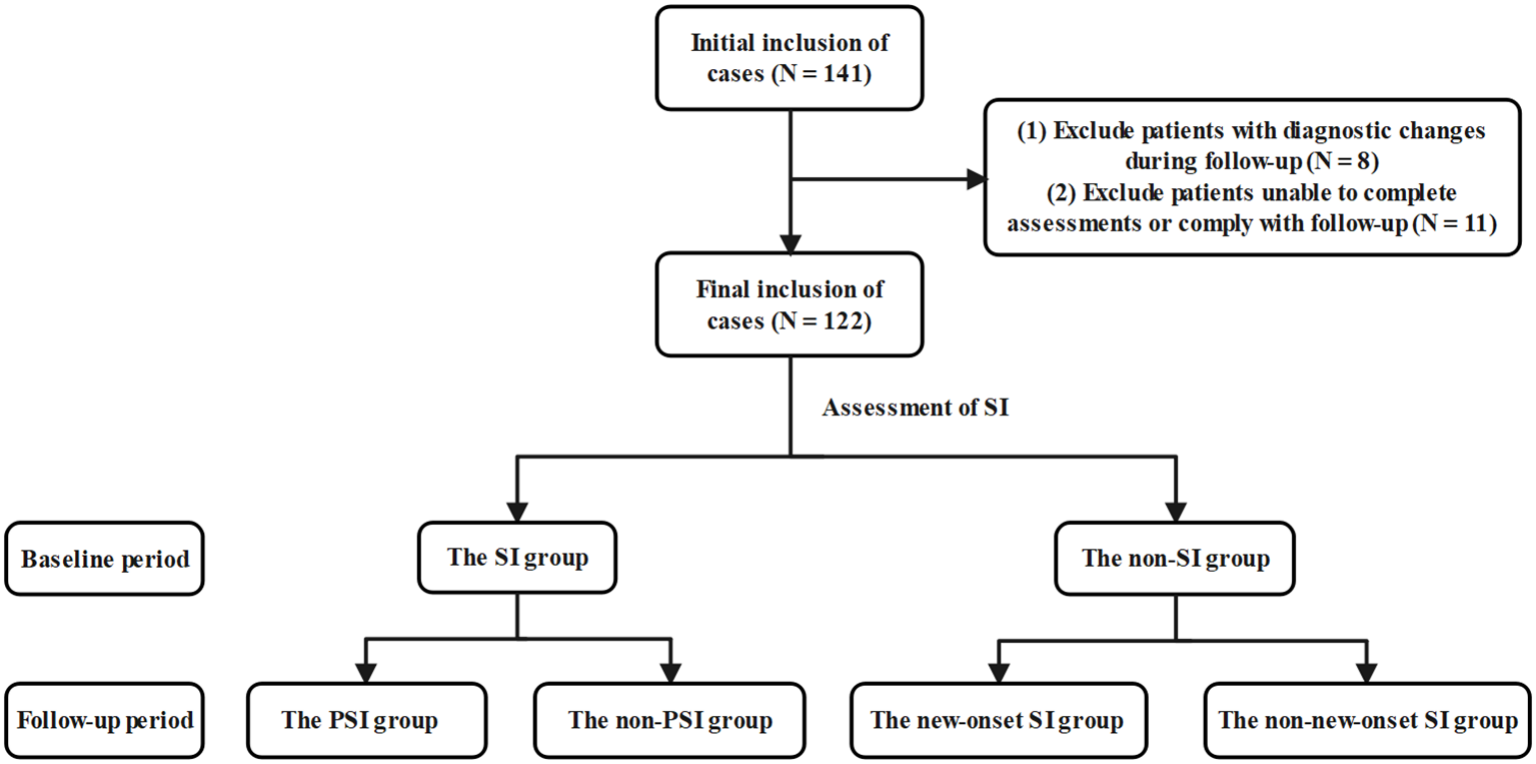
Flow diagram of patients selection.

This study included a total of 122 adolescents with MDD (**Figure 1**), among whom 87 were female (71.3%), with a mean age of 15.61 ± 1.57 years. Regarding SI, a total of 83 patients (68.0%) reported having SI, 24 patients (19.7%) exhibited PSI, and 25 patients (20.5%) experienced new-onset SI.

At the baseline period, the SI group exhibited significantly higher total scores on CTQ, CES-D, and TAS-20 compared with the non-SI group (all *P* < 0.05). Among them, the SI group had significantly higher scores than the non-SI group on the CTQ scale for emotional abuse (*P* < 0.001) and emotional neglect (*P* = 0.009), on the CES-D scale for depressed affect (*P* = 0.019) and interpersonal difficulties (*P* = 0.020), and on the TAS-20 scale for difficulty identifying feelings (*P* = 0.050) and externally oriented thinking (*P* = 0.021) (**Table 1**). In summary, the above differences indicated that adolescents with MDD accompanied by SI exhibited more severe experiences of childhood trauma, heightened depressive symptoms, and increased levels of alexithymia.

**Table 1:** Comparison of socio-demographic and clinical characteristics between the SI group and the non-SI group in adolescents with MDD.

Variables	Total sample (n=122)	SI (n=58)	Non-SI (n=64)	*t/Z/χ^2^*	*P*
Age (years), mean (SD)	15.61 (1.57)	15.59 (1.44)	15.63 (1.69)	-0.136	0.892
BMI (kg/m^2^), mean (SD)	20.93 (3.88)	21.60 (4.46)	20.32 (3.19)	1.835	0.069
Age at onset (years), mean (SD)	13.85 (1.84)	13.67 (1.84)	14.02 (1.83)	-1.032	0.304
Duration of illness (months), median (P_25_, P_75_)	18.00 (9.75, 36.00)	24.00(12.00, 39.00)	15.00 (6.75, 24.00)	-1.745	0.081
Gender				0.066	0.798
Male, n (%)	35 (28.69)	16(27.59)	19 (29.69)		
Female, n (%)	87 (71.31)	42 (72.41)	45 (70.31)		
Single-child family				1.509	0.219
Yes, n (%)	56 (45.90)	30 (51.72)	26 (40.63)		
No, n (%)	66 (57.10)	28 (48.28)	38 (59.38)		
NSSI				1.043	0.307
Yes, n (%)	72 (59.02)	37 (63.79)	35 (54.69)		
No, n (%)	50 (40.98)	21 (36.21)	29 (45.31)		
Antidepressants				0.532	0.828
None, n (%)	31 (25.41)	14 (24.14)	17 (26.56)		
SSRIs, n (%)	83 (68.03)	41 (70.69)	42 (65.63)		
Others, n (%)	8 (6.56)	3 (5.17)	5 (7.81)		
CES-D score, mean (SD)	36.35 (11.97)	39.12 (10.27)	33.84 (12.90)	2.482	0.014
Depressed affect	14.51 (5.64)	15.76 (4.86)	13.38 (6.08)	2.377	0.019
Positive affect	8.94 (2.69)	9.43 (2.53)	8.50 (2.77)	1.932	0.056
Somatic complaints	10.13 (3.96)	10.72 (3.56)	9.59 (4.24)	1.586	0.115
Interpersonal difficulties	2.77 (2.00)	3.21 (1.83)	2.38 (2.07)	-2.333	0.020
CTQ score, mean (SD)	49.52 (11.88)	53.21 (11.18)	46.19 (11.59)	3.397	0.001
Emotional abuse	11.52 (4.42)	12.97 (4.42)	10.22 (4.03)	3.590	<0.001
Physical abuse	7.16 (2.50)	7.55 (2.46)	6.80 (2.50)	1.680	0.095
Sexual abuse	5.80 (2.08)	5.97 (2.30)	5.64 (1.86)	0.860	0.391
Emotional neglect	14.83 (5.09)	16.09 (5.20)	13.69 (4.75)	2.663	0.009
Physical neglect	10.22 (3.22)	10.64 (3.32)	9.84 (3.11)	1.365	0.175
TAS-20 score, mean (SD)	68.19 (7.73)	70.24 (7.60)	66.33 (7.43)	2.874	0.005
Difficulty identifying feelings	26.74 (4.66)	27.60 (4.73)	25.95 (4.48)	1.978	0.050
Difficulty describing feelings	18.33 (2.77)	18.74 (2.71)	17.95 (2.80)	1.579	0.117
Externally oriented thinking	23.12 (3.53)	23.90 (3.56)	22.42 (3.38)	2.347	0.021

SI, suicidal ideation; BMI, body mass index; NSSI, non-suicidal self-injury; SSRIs, selective serotonin reuptake inhibitors; CES-D, the center for epidemiological studies depression scale; CTQ, the childhood trauma questionnaire; TAS-20, the toronto alexithymia scale.

SD, standard deviation; Bolded *P* value: < 0.05.

At the one-year follow-up period, the proportion of single-child family, the proportion of NSSI, the total score of TAS-20, the score of interpersonal difficulties and the score of difficulty identifying feelings in the PSI group were significantly higher than those in the non-PSI group (all *P* < 0.05). In addition, the proportion of NSSI, the total score of CES-D, the score of depressed affect and the score of positive affect in the new-onset SI group were significantly higher than those in the non- new-onset SI group (all *P* < 0.05) (**Tables 2**, **3**). In conclusion, adolescents with PSI exhibited significantly higher rates of single-child family, NSSI and alexithymia, while those with new-onset SI showed a significant increase in NSSI risk and depressive symptoms.

**Table 2:** Comparison of socio-demographic and clinical characteristics between the PSI group and the non-PSI group in adolescents with MDD.

Variables	PSI (n=24)	Non-PSI (n=34)	*t/Z/χ^2^*	*P*
Age (years), mean (SD)	15.25 (1.33)	15.82 (1.49)	1.511	0.136
BMI (kg/m^2^), mean (SD)	21.73 (4.92)	21.51 (4.17)	-0.181	0.857
Age at onset (years), mean (SD)	13.25 (1.92)	13.97 (1.75)	1.485	0.143
Duration of illness (months), median (P_25_, P_75_)	24.00 (12.00, 36.00)	17.00 (8.50, 48.00)	-0.45	0.653
Gender			0.935	0.334
Male, n (%)	5 (20.83)	11 (32.35)		
Female, n (%)	19 (79.17)	23 (67.65)		
Single-child family			5.987	0.014
Yes, n (%)	17 (70.83)	13 (38.24)		
No, n (%)	7 (29.17)	21 (61.76)		
NSSI			4.189	0.041
Yes, n (%)	19 (79.17)	18 (52.94)		
No, n (%)	5 (20.83)	16 (47.06)		
Antidepressants			1.722	0.516
None, n (%)	7 (29.17)	7 (20.59)		
SSRIs, n (%)	15 (62.50)	26 (76.47)		
Others, n (%)	2 (8.33)	1 (2.94)		
CES-D score, mean (SD)	40.50 (10.92)	38.15 (9.84)	-0.857	0.395
Depressed affect	15.63 (5.41)	15.85 (4.51)	0.175	0.862
Positive affect	9.88 (2.37)	9.12 (2.63)	-1.126	0.265
Somatic complaints	11.21 (3.89)	10.38 (3.32)	-0.869	0.389
Interpersonal difficulties	3.79 (1.72)	2.79 (1.82)	-2.471	0.013
CTQ score, mean (SD)	53.83 (12.73)	52.76 (10.13)	-0.356	0.723
Emotional abuse	12.88 (4.87)	13.03 (4.15)	0.130	0.897
Physical abuse	7.63 (2.65)	7.50 (2.35)	-0.189	0.851
Sexual abuse	6.08 (2.06)	5.88 (2.48)	-0.325	0.746
Emotional neglect	15.71 (5.80)	16.35 (4.80)	0.462	0.646
Physical neglect	11.54 (3.31)	10.00 (3.22)	-1.776	0.081
TAS-20 score, mean (SD)	72.92 (7.17)	68.35 (7.42)	-2.339	0.023
Difficulty identifying feelings	29.17 (4.58)	26.50 (4.57)	-2.185	0.033
Difficulty describing feelings	19.25 (2.40)	18.38 (2.88)	-1.208	0.232
Externally oriented thinking	24.50 (3.04)	23.47 (3.88)	-1.086	0.282

PSI, persistent suicidal ideation; BMI, body mass index; NSSI, non-suicidal self-injury; SSRIs, selective serotonin reuptake inhibitors; CES-D, the center for epidemiological studies depression scale; CTQ, the childhood trauma questionnaire; TAS-20, the toronto alexithymia scale.

SD, standard deviation; Bolded *P* value: < 0.05.

**Table 3:** Comparison of socio-demographic and clinical characteristics between the new-onset SI group and the non-new-onset SI group in adolescents with MDD.

Variables	New-onset SI (n=25)	Non-new-onset SI (n=39)	*t/Z/χ^2^*	*P*
Age (years), mean (SD)	15.56 (1.58)	15.67 (1.77)	-0.245	0.807
BMI (kg/m^2^), mean (SD)	19.76 (3.74)	20.68 (2.78)	-1.125	0.265
Age at onset (years), mean (SD)	13.80 (2.08)	14.15 (1.66)	-0.752	0.455
Duration of illness (months), median (P_25_, P_75_)	18.00 (12.00, 24.00)	12.00 (6.00, 30.00)	-0.007	0.994
Gender			0.783	0.376
Male, n (%)	9 (36.00)	10 (25.64)		
Female, n (%)	16 (64.00)	29 (74.36)		
Single-child family			2.201	0.138
Yes, n (%)	13 (52.00)	13 (33.33)		
No, n (%)	12 (48.00)	26 (66.67)		
NSSI			7.520	0.006
Yes, n (%)	19 (76.00)	16 (41.03)		
No, n (%)	6 (24.00)	23 (58.97)		
Antidepressants			0.990	0.719
None, n (%)	6 (24.00)	11 (28.21)		
SSRIs, n (%)	18 (72.00)	24 (61.54)		
Others, n (%)	1 (4.00)	4 (10.26)		
CES-D score, mean (SD)	39.08 (11.69)	30.49 (12.65)	2.73	0.008
Depressed affect	15.76 (5.50)	11.85 (6.00)	2.629	0.011
Positive affect	9.84 (2.10)	7.64 (2.83)	3.336	0.001
Somatic complaints	10.60 (4.35)	8.95 (4.10)	1.536	0.130
Interpersonal difficulties	2.88 (2.22)	2.05 (1.92)	-1.508	0.131
CTQ score, mean (SD)	48.04 (9.170	45.00 (12.87)	1.024	0.310
Emotional abuse	10.64 (3.34)	9.95 (4.44)	0.708	0.481
Physical abuse	7.24 (2.89)	6.51 (2.20)	1.140	0.259
Sexual abuse	5.40 (1.04)	5.79 (2.24)	-0.825	0.413
Emotional neglect	14.72 (4.63)	13.03 (4.77)	1.402	0.166
Physical neglect	10.04 (2.39)	9.72 (3.52)	0.436	0.664
TAS-20 score, mean (SD)	67.72 (7.00)	65.44 (7.65)	1.204	0.233
Difficulty identifying feelings	27.00 (4.35)	25.28 (4.50)	1.510	0.136
Difficulty describing feelings	18.52 (2.63)	17.59 (2.87)	1.305	0.197
Externally oriented thinking	22.20 (3.76)	22.56 (3.14)	-0.418	0.677

SI, suicidal ideation; BMI, body mass index; NSSI, non-suicidal self-injury; SSRIs, selective serotonin reuptake inhibitors; CES-D, the center for epidemiological studies depression scale; CTQ, the childhood trauma questionnaire; TAS-20, the toronto alexithymia scale.

SD, standard deviation; Bolded *P* value: < 0.05.

### 3.2 Multivariate logistic stepwise regression analysis of SI in adolescents with MDD

At the baseline period, Model 1 indicated that CTQ score (*OR* = 1.055, 95%*CI*: 1.021 - 1.091, *P* = 0.002) was a risk factor for SI in adolescents with MDD. In Model 2, the score of emotional abuse (*OR* = 1.161, 95%*CI*: 1.057 - 1.274, *P* = 0.002) and the score of externally oriented thinking (*OR* = 1.120, 95%*CI*: 1.002 - 1.251, *P* = 0.046) were identified as risk factors for SI.

At the follow-up period of the baseline SI group, Model 1 showed that single-child family (*OR* = 3.969, 95%*CI:* 1.227 - 12.839, *P* = 0.021) and TAS-20 score (*OR* = 1.091, 95%*CI*: 1.006 - 1.184, *P* = 0.035) were risk factors for PSI in adolescents with MDD. In Model 2, single-child family (*OR* = 3.869, 95%*CI*: 1.212 - 12.345, *P* = 0.022) and the score of difficulty identifying feelings (*OR* = 1.134, 95%*CI:* 1.000 - 1.287, *P* = 0.050) were risk factors for PSI.

At the follow-up period of the baseline non-SI group, Model 1 showed that NSSI (*OR* = 4.552, 95%*CI*: 1.488 - 13.921, *P* = 0.008) was a risk factor for new-onset SI in adolescents with MDD. In Model 2, the score of positive affect (*OR* = 1.424, 95%*CI*: 1.125-1.804, *P* = 0.003) was a risk factor for new-onset SI (**Table 4**). In addition, multicollinearity among the variables in this study was also assessed by calculating the variance inflation factor (VIF). All VIF values were less than 5.0, indicating that the multicollinearity was within acceptable limits.

**Table 4:** Multivariate logistic stepwise regression analysis of SI in adolescents with MDD.

Models	Variables	*OR*	95% *CI*	*P*
SI				
Model 1 ^a^	CTQ score	1.055	1.021 - 1.091	**0.002**
Model 2 ^b^	Emotional abuse	1.161	1.057 - 1.274	**0.002**
	Externally oriented thinking	1.120	1.002 - 1.251	**0.046**
PSI				
Model 1	Single-child family	3.969	1.227 - 12.839	**0.021**
	TAS-20 score	1.091	1.006 - 1.184	**0.035**
Model 2	Single-child family	3.869	1.212 - 12.345	**0.022**
	Difficulty identifying feelings	1.134	1.000 - 1.287	**0.050**
New-onset SI				
Model 1	NSSI	4.552	1.488 - 13.921	**0.008**
Model 2	Positive affect	1.424	1.125 - 1.804	**0.003**

SI, suicidal ideation; PSI, persistent suicidal ideation; CTQ, the childhood trauma questionnaire; TAS-20, the toronto alexithymia scale; NSSI, non-suicidal self-injury.

OR, odds ratio; CI, confidence interval; Bolded *P* value: < 0.05.

^a^, the total score of CES-D, CTQ and TAS-20 involved in the regression model; ^b^, subscale scores of CES-D, CTQ and TAS-20 involved in the regression model.

In summary, at the baseline period, the CTQ score and its emotional abuse subscale, along with externally oriented thinking on the TAS-20, independently predicted the presence of suicidal ideation. At the follow-up period, the main drivers of PSI included single-child family and TAS-20 score, among which the dimension of difficulty identifying feelings was particularly significant. NSSI behavior and the absence of positive affect were the main predictors of new-onset SI.

## 4 Discussion

Given the strong predictability of SI for suicidal behavior, an in-depth study of its longitudinal developmental process and associated influencing factors is of great significance for reducing suicide risk. This study demonstrated that the prevalence of PSI in adolescents with MDD was 19.7%, and the prevalence of new-onset SI was 20.5%. The prevalence of PSI in this study was significantly higher than that reported in the general adolescent population in China. A previous study on Chinese teenagers reported that in the general student population, the prevalence of individuals with PSI was only 1.8%, and approximately two-fifths of these individuals indicated that they had further suicidal plans and attempts (5). Furthermore, in a study encompassing 17 countries, 60% of individuals experienced the transition from SI to a first suicide attempt within the first year following the onset of SI (39). These findings suggest that clinicians need to identify high-risk groups at an early stage and intervene in a timely manner to enhance the effectiveness of preventive measures.

At the baseline period, this study found that the total score of the CES-D, CTQ, and TAS-20 were significantly higher in the SI group than in the non-SI group, consistent with previous studies. Firstly, an increasing number of studies have confirmed a direct positive correlation between the severity of depressive symptoms and SI. A study on SI in Chinese adolescents with MDD indicated that the severity of depression could serve as a significant predictor of SI in this population (4). A follow-up study further demonstrated that changes in depressive symptoms could predict subsequent changes in SI, thereby supporting the temporal impact of the severity of depressive symptoms on SI (16). Secondly, CM is an important predictor of SI. A meta-analysis demonstrated that CM can increase an individual’s risk of SI by 2.3 times, and the earlier the CM occurs, the higher the risk (40). On this basis, the study found that emotional abuse significantly influences SI in Chinese adolescents through a cascade of stress-related mediators (41). Similarly, a study on CM and SI indicated that emotional abuse influenced SI via separation-related mediators, emotional neglect influenced SI via despair-related mediators, and sexual abuse had a direct impact on SI (42). These findings suggest the necessity of focusing on the potential risks associated with different types of CM and implementing targeted interventions within suicide prevention efforts. Finally, a cross-sectional study indicated that alexithymia was a significant predictor of SI in patients with MDD, and this association remains significant even after controlling for depressive symptoms (43). Further research found that adolescents with MDD who exhibited pronounced extroverted thinking were more likely to engage in negative behaviors due to their inability to effectively manage negative emotions (44). Therefore, improving depressive symptoms, optimizing the family environment, and enhancing emotional regulation skills may help adolescents with MDD to improve their mental health status, reduce the occurrence of SI and behavior, and thereby promote the recovery of social functioning.

At the follow-up period, single-child family, the total score of TAS-20, and the score of difficulty identifying feelings were all identified as risk factors for PSI in adolescents with MDD, suggesting a possible synergy between family environment and emotional regulation deficits. Previous studies showed that single-child family was one of the key risk factors for SI in adolescents. A study found through logistic regression analyses that the background of single-child family increased the risk of SI in adolescents by more than four times (45). This might be related to certain unique psychological experiences encountered by children in single-child family during their developmental trajectory. For instance, children from single-child family often experienced a higher degree of loneliness, and chronic feelings of loneliness were closely associated with SI (46). These results suggest that the uniqueness of children from single-child family in terms of family support, psychological stress, and emotional regulation may be an important factor for the persistence of SI. Alexithymia, a stable personality trait characterized by difficulty in recognizing and describing one’s own emotions, is closely associated with difficulties in emotion regulation (47). A study on SI proposed that individuals with a lack of emotional awareness, inadequate coping and regulation strategies, and an inability to accept their emotions were more likely to report PSI (48). This discovery was in accordance with a study on the biosocial theory, which posited that difficulty in emotion regulation was a key factor in maintaining suicidal tendencies (49). Specifically, a meta-analysis revealed a significant association between SI and alexithymia, particularly difficulty identifying feelings (50). Furthermore, a domestic study also found that difficulty in emotion recognition was not only an independent risk factor for SI, but also indirectly affected the generation and persistence of SI by intensifying perceived stress and depressive symptoms (26). Therefore, in patients with MDD, the disorder of emotion recognition and expression can lead to the long-term accumulation of negative emotions, thereby intensifying the persistence of SI. However, although the effect size of the TAS-20 score reached statistical significance, its corresponding OR close to 1, indicating that its clinical significance requires further validation. Future studies should employ multicenter, large-sample designs to reassess its effect size in order to clarify its actual clinical utility.

In addition, this study found that NSSI and CES-D (positive affect) score could be used as risk factors for predicting new-onset SI, which was basically consistent with previous studies. There is a significant association between NSSI and SI, which has a longitudinal predictive effect. Specifically, a longitudinal study of adolescents showed that NSSI at baseline significantly predicted subsequent SI, which in turn reinforced NSSI behavior, forming a bidirectional cycle (51). Moreover, the severity and persistence of NSSI had a stratified effect on suicide risk. Individuals with a high frequency or persistence of NSSI exhibited more severe SI and behavior, whereas a decrease in the frequency of NSSI was accompanied by a parallel decrease in SI (52, 53). This association may have stemmed from the negative self-perception of individuals with NSSI, such as low self-esteem, low self-efficacy, and excessive self-criticism (54, 55). These traits were not only closely related to NSSI, but may also have further strengthened SI, thereby forming a vicious cycle. A study further indicated that NSSI might be a maladaptive coping strategy. In the short term, it reduced SI, but in the long term, it increased the risk of suicide (56). Therefore, NSSI is not only a risk marker for SI, but may also influence long-term changes in suicide risk through complex mechanisms. In the CES-D, the reduction of positive affect is a core dimension of depressive symptoms. This subscale is reverse-scored, and the higher scores are associated with fewer positive affect experienced by individuals and more severe depressive symptoms. Previous studies indicated that the reduction of positive affect might serve as one of the important mechanisms underlying the development of SI and behavior among adolescents. A prospective study found that lower levels of positive affect still significantly predicted the risk of suicide-related events in adolescents at 6 months, after controlling for levels of depression, world-aversion, and other potential confounders (57). A study demonstrated that a lower level of positive affect in an individual’s daily life was a significant risk factor for SI the following day (58). Furthermore, a randomized controlled trial indicated that the reduction of positive affect may increase the risk of SI among teenagers by enhancing their feelings of depression and despair (17). In conclusion, NSSI and the reduction of positive affect both reflect that individuals lack effective coping strategies when facing negative emotions and life stress, thereby increasing the risk of SI in the future.

This study systematically elucidated the key predictors of persistent or new-onset SI in adolescents with MDD, and provided an empirical basis for the development of evidence-based screening indicators and early intervention programs. Based on these findings, it is recommended to introduce a brief and effective screening tool in routine clinical assessments and early identification system in school/community, focusing on alexithymia, single-child family, NSSI behaviors and changes in positive affect. Early interventions should be implemented for high-risk individuals identified through screening to enhance their emotional regulation, reduce the incidence of NSSI behaviors and enhance experiences of positive affect. Meanwhile, families, schools, and society should collaborate to establish a multi-layered support system and optimize psychological intervention measures, thereby effectively reducing the risks of the emergence and persistence of SI and jointly safeguarding the mental health of adolescents.

This study also had certain limitations: (1) This study was exploratory in nature, with a relatively small sample size and limited statistical power. Furthermore, the study population was entirely recruited from Anhui Province, which may limit the generalizability of the findings. Therefore, future multicenter, large-sample prospective clinical studies are needed to further investigate this issue, thereby enhancing the statistical power and reliability of the findings. (2) In this study, SI was assessed only at baseline and one-year follow-up. Future studies will add additional assessment time points to more accurately capture the trajectory of SI, thereby improving the accuracy and reliability of the study data. (3) This study did not adequately consider all of the potential confounders that may influence SI in adolescents, such as biological, genetic, and environmental factors. Future studies will address these limitations by including a wider range of variables in their analyses.

In conclusion, this study found that PSI in adolescents with MDD was closely associated with single-child family and alexithymia, whereas new-onset SI was influenced by NSSI and reduced positive affect. These factors may further promote the occurrence and persistence of SI by exacerbating difficulties in emotion regulation, negative self-perception, and depressive symptoms.

## Data Availability

The raw data supporting the conclusions of this article will be made available by the authors, without undue reservation.
